# Household expenditures on pneumonia and diarrhoea treatment in Ethiopia: a facility-based study

**DOI:** 10.1136/bmjgh-2016-000166

**Published:** 2017-01-18

**Authors:** Solomon Tessema Memirie, Zewdu Sisay Metaferia, Ole F Norheim, Carol E Levin, Stéphane Verguet, Kjell Arne Johansson

**Affiliations:** 1 Department of Global Public Health and Primary Care, University of Bergen, Bergen, Norway; 2 ElnaMaZ Public Health Consulting, Addis Ababa, Ethiopia; 3 Department of Global Health, University of Washington, Seattle, Washington, USA; 4 Department of Global Health and Population, Harvard T. H. Chan School of Public Health, Boston, Massachusetts, USA

## Abstract

**Background:**

Out-of-pocket (OOP) medical payments can lead to catastrophic health expenditure and impoverishment. We quantified household OOP expenditure for treatment of childhood pneumonia and diarrhoea and its impact on poverty for different socioeconomic groups in Ethiopia.

**Methods:**

This study employs a mix of retrospective and prospective primary household data collection for direct medical and non-medical costs (2013 US$). Data from 345 pneumonia and 341 diarrhoea cases (0–59 months of age) were collected retrospectively through exit interviews from 35 purposively sampled health facilities in Ethiopia. Prospective 2-week follow-up interviews were conducted at the household level using a structured questionnaire.

**Results:**

The mean total medical expenditures per outpatient visit were US$8 for pneumonia and US$6 for diarrhoea, while the mean for inpatient visits was US$64 for severe pneumonia and US$79 for severe diarrhoea. The mean associated direct non-medical costs (mainly transport costs) were US$2, US$2, US$13 and US$20 respectively. 7% and 6% of the households with a case of severe pneumonia and severe diarrhoea, respectively, were pushed below the extreme poverty threshold of purchasing power parity (PPP) US$1.25 per day. Wealthier and urban households had higher OOP payments, but poorer and rural households were more likely to be impoverished due to medical payments.

**Conclusions:**

Households in Ethiopia incur considerable costs for the treatment of childhood diarrhoea and pneumonia with catastrophic consequences and impoverishment. The present circumstances call for revisiting the existing health financing strategy for high-priority services that places a substantial burden of payment on households at the point of care.

**Key questions**

**What is already known about this topic?**

- Out-of-pocket (OOP) medical payments can lead to catastrophic health expenditure and impoverishment. Studies on household healthcare cost of pneumonia and diarrhoeal disease among children under five are scarce in sub-Saharan African countries and are non-existent in Ethiopia.

**What are the new findings?**

- Better estimates of the current household OOP medical payments for pneumonia and diarrhoea treatment in developing countries allow for more precision in estimating the expected poverty impact of health interventions such as vaccines, independent of the interventions' health impact.

**Recommendations for policy**

- The study is on OOP medical expenses that may not have a direct impact on clinical practice. However, given the fact that OOP payments for the treatment of pneumonia or diarrhoea are high and the major cost driver being medication might influence the choice of generic drugs over brands.

## Introduction

In low-income and middle-income countries, diarrhoea and respiratory infections are the most common causes of childhood illnesses and healthcare visits. Similarly, severe cases of diarrhoea and pneumonia are among the most common reasons for hospital admission of children. Childhood pneumonia and diarrhoea are the leading causes of death globally and in Ethiopia.^1^ ^2^

Illnesses impose a huge economic burden on individuals and families. Direct payments for healthcare can have negative consequences for families, including pushing families into poverty or further into deeper poverty. User fees exacerbate inequity, as poor people are more likely to reduce service usage and become impoverished from the effects of catastrophic health expenditures (CHE)—defined as household's financial contributions to the health system exceeding 40% of income remaining after subsistence needs have been met.^3^

The 2005 healthcare financing reform in Ethiopia allowed public health facilities to collect, retain and use the revenues and user fees that they generate from different sources, as an addition to the government budget, for improving the quality of health services.^4^ The retained revenues generated from user fees covered 56% of the total health budget for health centres in the year 2011/2012.^5^ A system of fee waivers and exemptions was part of the reform. Though preventive services (eg, immunisation, prenatal care, etc) are delivered freely at public health facilities, curative child health services are not provided free of charge in public health centres and public hospitals.

Attainment of universal health coverage (UHC) is a central theme of the Sustainable Development Goals.^6^ To achieve UHC, countries should address all three dimensions of the cube: (1) Whom to include first? (2) Which services to cover? (3) Proportion of the costs covered. The World Health Report identifies continued reliance on direct payments, including user fees, as by far the greatest obstacle to the attainment of UHC.^7^ Despite fee waivers for preventive health services, the OOP expenditures for curative care for children are a burden in Ethiopia, accounting for close to 50% of total child healthcare expenditures in 2010/2011.^8^

Studies on household medical expenses of pneumonia and diarrhoea among children under five are scarce in sub-Saharan African countries. Two studies, one in Kenya and another in Zambia, have examined medical costs of pneumonia treatment.^9^ ^10^ The study in Kenya was hospital based while the study in Zambia involved one health centre, so neither study was representative of nationwide disease costs of inpatient and outpatient pneumonia treatment. Rheingans *et al*,^11^ in their study in three African countries, concluded that diarrhoea episodes resulted in substantial economic cost. To date, no studies have measured healthcare costs of pneumonia or diarrhoea in Ethiopia.

Ethiopia has recently introduced pneumococcal conjugate vaccine and rotavirus vaccine as part of the basic vaccine programme.^12^ Reduction in new cases of pneumonia and diarrhoea may offer protection against impoverishment and OOP expenditures for such diseases. Better estimates of the current household OOP expenses allow for more precision in estimating the expected poverty impact of these new vaccines, independent of the interventions' health benefits.^13^ ^14^

The objectives of this study are to: (1) estimate and characterise household OOP expenses for an episode of childhood diarrhoea and pneumonia by type and level of care; (2) assess the extent to which OOP expenses for diarrhoea and pneumonia contribute to household impoverishment and (3) examine the distribution of household OOP expenses across wealth quintiles and by place of residence.

## Methods

We conducted a descriptive facility-based cost study of diarrhoeal disease and all-cause pneumonia in children under five in Ethiopia from the household (patient) perspective. OOP expenses were measured in terms of local currency and converted to US dollars (US$). The average 2013 exchange rate of 18.6 Ethiopian Birr (ETB) to US$1 was used.^15^

### Study area and population

Ethiopia is the second most populous country in Africa with an estimated 94 million inhabitants.^16^ A majority of the Ethiopian population lives in rural areas (84%), and the population pyramid remains quite young: 44% are under 15 years of age.^17^ At present, Ethiopia is administratively structured into nine national regional states—Oromia, Amhara, Southern Nations Nationalities and People Region (SNNPR), Tigray, Benishangul-Gumuz, Gambella, Afar, Somali and Harari—and two city administrations, that is, Addis Ababa City Administration and Dire Dawa City Council. In spite of rapid economic development in the last decade, at an average annual growth rate of 11% per year, Ethiopia remains one of the poorest countries in Africa with annual per capita earnings of about US$550, which is well below the sub-Saharan African average of US$1640.^18^ ^19^

**Figure 1:**
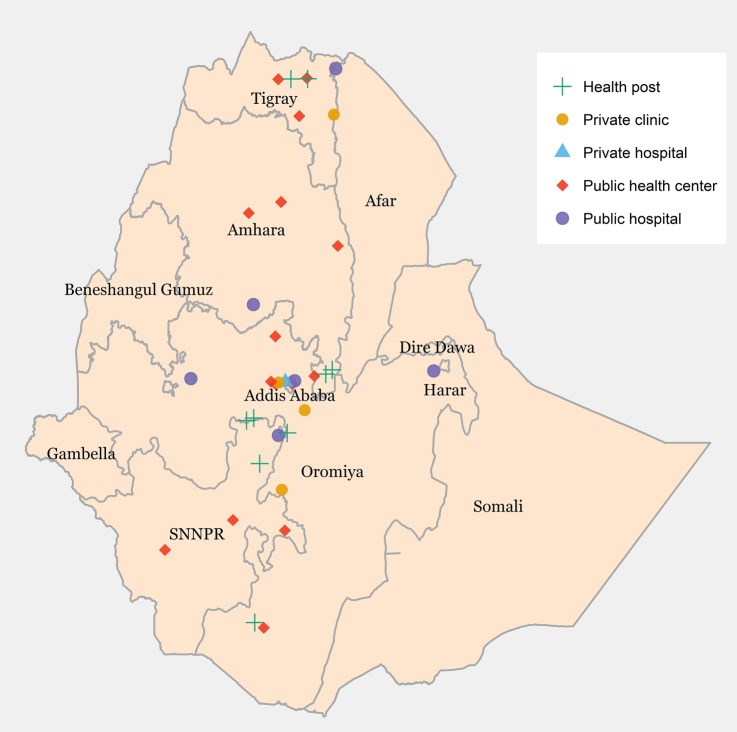
Distribution of health facilities included in the study.

The study population was individuals seeking treatment at health facilities in four major regions (Oromia, Amhara, SNNPR and Tigray) and Addis Ababa city administration (the capital city) in Ethiopia (figure 1). These regions were selected because they are home to 90% of the Ethiopian population and are ethnically and culturally diverse. Furthermore, 90% of the health centres and 81% of the public hospitals are located in these regions and the capital city.^20^ Public healthcare delivery in Ethiopia consists of a three-tier system.^21^ The primary healthcare (PHC) unit is the first level which is composed of a Health Center, five satellite Health Posts and Primary Hospital serving an average population of 100 000. The secondary care level comprised general hospitals. Each general hospital provides inpatient and ambulatory services to an average of 1 million people. Tertiary care is provided in specialised hospitals each serving an average of 5 million people. Among those who sought care in health facilities for a complaint of cough or diarrhoea, 25% and 23%, respectively, visited the private health sector (Demographic and Health Survey (DHS), 2011).^22^

### Study sites and sample selection

Data were collected from individuals seeking services from a sample of 6 public hospitals, 15 public health centres, 9 health posts and 5 private health facilities (a total of 35 health facilities).

We used convenience sampling to select facilities after stratifying them based on level of care (primary to tertiary), urban/rural^i^ location^23^ and implementation of the integrated management of childhood illnesses (IMCI) strategy. DHS 2011 disaggregates the type of facilities visited for cases of acute respiratory infection or diarrhoea. We used these data as a reference to allocate the number of cases enrolled in the study by type of facility (table 1).

**Table 1:** Distribution of cases by type of facility visited in the five regions included in the study

	Regions									
	Oromia		Amhara		SNNPR		Tigray		Addis Ababa	
Type of health facility	No. of health facilities	No. of cases	No. of health facilities	No. of cases	No. of health facilities	No. of cases	No. of health facilities	No. of cases	No. of health facilities	No. of cases
Public hospital	2	51	1	25	1	30	1	26	1	28
Health centre	4	95	3	72	3	45	3	90	2	66
Health post	3	29	2	20	2	20	2	20	–	–
Private clinic/hospital	1	21	1	8	1	10	1	10	1	20
Total	10	196	7	125	7	105	7	146	4	114

SNNPR, Southern Nations Nationalities and People Region.

We included children 0–59 months of age with a clinical diagnosis of pneumonia or diarrhoea but without other illnesses. On the basis of two previous cost studies,^24^ ^25^ we calculated that 65 patients in each wealth quintile would allow reporting of results, suggesting a mean difference of at least 3.0 ETB across successive wealth quintiles with a SD of 6.1 ETB at 95% level of confidence and a power of 80. Hence, we aimed to collect data from a sample consisting of 375 patients (325 plus 15% non-response) with a diagnosis of pneumonia or diarrhoea. We planned to include 33 severe pneumonia and 33 severe diarrhoea cases (10% of diarrhoea and 10% of pneumonia cases) admitted for inpatient care in hospitals. Outpatient cases were enrolled consecutively when an IMCI-trained clinician identified them as having either diarrhoea or pneumonia until the sample size quota was obtained. Similarly, severe cases of pneumonia or diarrhoea were consecutively enrolled from paediatric inpatient units after the physician in charge had confirmed the diagnosis of either severe pneumonia or severe diarrhoea.

### Data collection

This study employs a mix of retrospective and prospective primary household data collection. Data on direct medical costs (registration, diagnostic workup, medications and hospital bed), direct non-medical costs (transportation, food and drinks, lodging, etc) and parents’ time loss were collected through exit interview using a retrospective structured questionnaire. Furthermore, parents were asked whether they had used over-the-counter medications and/or had a visit to traditional healers before visiting the formal private or public sector. In order to ascertain recovery and estimate additional costs (families may incur additional healthcare expenses in relation to the current illness after leaving the facility), a prospective follow-up interview was conducted at the household level within 2 weeks of initial interview or discharge. The additional expenses may relate to having another visit (because they failed to improve or for follow-up) or costs related to injections or other costs. If additional costs were incurred, we included these costs in the calculation of total medical expenditures. To collect OOP expenses for the current illness episode, we used a reference period of 2 weeks (the time between initial and follow-up interviews), since pneumonia and diarrhoea episodes are usually acute and were likely to be resolved in the period.

Household consumption expenditure data were collected by asking caretakers for monthly estimates of amounts spent on food, housing, fuel, electricity, water, education and healthcare for the month preceding the survey. Whenever possible, household heads were involved in eliciting expenditures on specific items. We derived an estimate of annual household expenditures based on the monthly survey data. Households were also asked about the availability of durable consumer goods such as radio, television, refrigerator, bicycle, car/truck, motorbike, farm equipment and agricultural land. Caretakers’ time loss was estimated by adding the time spent seeking healthcare prior to outpatient consultation and/or admission and the duration of outpatient and/or inpatient stay.

An investigator visited each site, identified an IMCI-trained nurse (in hospitals and health centres) or IMCI-trained health extension worker (in health posts) and provided training on the use of data collection tools to ensure that data were accurate, complete and consistent across sites. In hospitals and health centres, the investigator observed the data collection process on at least one patient with either pneumonia or diarrhoea. Owing to the low case load at health post level, exit interviews could not yield the required results in a reasonable time. We therefore identified households that had accessed care from registers at the health posts after which data were collected through a visit to their dwellings. Data were collected for the period August–December, 2013. All study participants gave a written informed consent.

### Data analysis

To obtain direct medical expenses per case, we added up OOP payments for registration, diagnostic work-up, medications and hospital stay. Similarly, direct non-medical expenses per case were calculated by summing the OOP payments for transportation, food, lodging and other costs incurred in relation to treatment services sought and received. Total OOP expenditure per case was calculated as the sum of the direct medical and non-medical expenses. We did not estimate the economic value of productivity losses associated with caregiver's transport and health seeking time. The two accepted approaches to value time loss (human capital and friction cost approaches) use gross wages, which is less meaningful in an economy that is largely subsistence.^26^

We examined how household economic status, type of health facility, region and geographic locations (urban vs rural) were associated with OOP expenses incurred by households. We used the logarithmic transformation of OOP expenses because of the skew in the natural distribution of costs. We used linear regression model (after log transformation of OOP costs) to assess the effect of the predictor variables on the mean household OOP expenses. Logistic regression was used to identify the variables that were major drivers of differences in the rate of catastrophic head count among different wealth quintiles, by type of health facility visited and place of residence. P values of 0.05 or lower were deemed to be significant.

CHE to households associated with healthcare OOP expenses for pneumonia or diarrhoea was calculated by computing OOP expenditure incurred minus any reimbursements from third-party payers divided by annual household non-food expenditure (capacity to pay defined as effective income net of subsistence spending), following the WHO definition of CHE.^3^ More specifically, we defined capacity to pay (non-food expenditure) as total household expenditure net of food spending. One can better distinguish between the rich and the poor by using non-food expenditures than total expenditure.

We measured the incidence (catastrophic payment head count) of catastrophic expenditures.^27^ The measurement of this parameter is as follows: let P be OOP healthcare payment, x be total household expenditure and y be food expenditure, therefore x−y is the capacity to pay. Then, a household is said to have incurred catastrophic payments if P/(x−y), exceeds a specified threshold, z. The threshold represents the point at which families will have severe disruptions to their living standards due to healthcare spending. We used the WHO CHE threshold of healthcare payments of at least 40% of a household's capacity to pay. As childhood diarrhoea and pneumonia are usually acute conditions with shorter durations of illnesses, we opted to examine short-term and long-term impact of OOP healthcare costs on households for a single illness episode. To assess short-term impact, we used monthly capacity to pay as the denominator in the computation of CHE, while the annual estimate for capacity to pay was used as denominator to assess long-term impact.

To measure catastrophic head count in relation to capacity to pay, let us define an indicator T, which equals 1 if P_i_/(x_i_−y_i_)>z and zero otherwise. Then, an estimate of the catastrophic head count (H) measured at the household level (i) is given by1
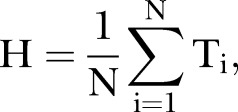
where N is the sample size.

Medical impoverishment was measured as the expected number of households that fell below the poverty threshold of US$1.25 due to OOP spending on healthcare. Poverty head count is the fraction of people living in poverty (fraction below the poverty line (PL)). First, we constructed a PL=3180 ETB using a PPP in 2013 of 6.97.^28^ Then, we computed the poverty head count as follows: let w_i_ be the per capita consumption expenditure of household i. An estimate of the poverty head count ratio without health payment deduction is2
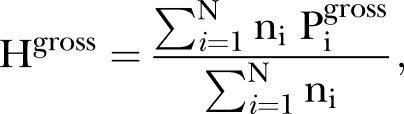
where 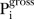
=1 if w_i_<PL and is 0 otherwise, n_i_ is the number of individuals in the household and N is the number of households in the sample. Then 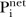 (the poverty head count after deducting healthcare payment from the per capita consumption expenditure) is computed as 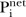
=1 if (w_i_−P_i_)<PL and is 0 otherwise.

The total household consumption expenditure and an adult equivalent (AE)^ii^ score (calculated based on the number and ages of household members) for each household were used to identify the economic quintile to which each study household belonged.^29^ Data were analysed using the statistical software package STATA (V.13).

### Ethical clearance

The study was approved by Regional committees for medical and health research ethics (REK) in Norway and Ethiopian Health and Nutrition Research Institute (EHNRI) scientific and ethical review committee.

## Results

### Sample characteristics

Of the 686 patients enrolled in the study (91% response rate), 303, 42, 309 and 32 were pneumonia, severe pneumonia, diarrhoea and severe diarrhoea cases, respectively. The mean age of patients was 1.7 years (95% CI 1.6 to 1.8 years). Details of sample characteristics are presented in table 2.

**Table 2:** Sample characteristics, by diagnosis

	Pneumonia	Severe pneumonia with inpatient care	Diarrhoea	Severe diarrhoea with inpatient care
No. of observations	303 (44%)	42 (6%)	309 (45%)	32 (5%)
Mean age in years (95% CI)	1.7 (1.5–1.8)	1.6 (1.0–2.1)	1.8 (1.7–2.0)	1.8 (1.3–2.4)
Sex distribution (% female)	48%	31%	51%	65%
Mean days of hospitalisation	–	4	–	3
Percentage of rural residents	37%	44%	41%	38%
Mean family size	4.89	5	4.82	4.88
Respondent (mother)	77%	44%	79%	50%
Respondent (father)	20%	56%	20%	50%
Mean age of the respondent in years (95% CI)	30 (30–31)	32 (29–35)	30 (29–31)	34.7 (30–39)
Respondents education (% with some secondary education)	34%	33%	29%	33%
Respondent's employment status (% in full time work)	38%	55%	37%	46%
Respondent's employment status (housewife)	50%	33%	51%	38%
Time spent by the respondent in relation to facility visit (hours)	8	96	6	78

### Costs to the household

Among the 686 patients enrolled in the study, 631 had complete data on costs incurred for the treatment of their current illness and on household consumption expenditures. Data on household consumption expenditure were missing for 55 study participants. We were able to reach 530 households for follow-up interviews to ascertain and record additional expenses incurred. We assumed that no additional expenses were incurred for the unreached households. We used data on these 631 cases for further cost analysis.

The mean OOP direct medical expenses (in 2013 US$) were US$6 and US$5 for outpatient pneumonia and diarrhoea services, respectively. Average OOP expenses were higher for inpatient services at US$51 for severe pneumonia and US$59 for severe diarrhoea. Medication costs accounted for the major share (60%) of direct medical costs. For inpatient care, the second largest expense was the bed charge, constituting 28% of direct medical costs. Diagnostic investigations covered 16% of direct medical costs. The average associated direct non-medical expenses (mainly transport costs) for pneumonia, diarrhoea, severe pneumonia and severe diarrhoea were US$2, US$2, US$13 and US$20, respectively. A breakdown of the direct medical and non-medical costs incurred by households is detailed in table 3.

**Table 3:** Mean (SD) medical expenditure in US$ per disease episode by cost type and diagnosis

	Diagnosis			
Cost type	Pneumonia	Diarrhoea	Severe pneumonia with inpatient care	Severe diarrhoea with inpatient care
Transportation	0.97 (2.22)	0.99 (3.30)	6.25 (7.66)	9.64 (11.02)
Registration/consultation	0.82 (1.76)	0.71 (1.45)	2.15 (2.72)	2.18 (2.64)
Laboratory	1.20 (3.48)	0.88 (2.36)	7.34 (12.94)	10.22 (17.05)
Medicines and supplies	4.27 (6.42)	3.02 (5.28)	28.53 (30.78)	28.89 (33.86)
Hospital bed	–	–	12.69 (15.37)	17.62 (32.96)
Traditional healer visit*	0.11 (0.56)	0.12 (0.90)	–	1.15 (4.47)
Other†	0.60 (2.31)	0.48 (2.09)	6.81 (6.35)	9.58 (12.61)
DMC‡	6.30 (10.51)	4.65 (8.43)	50.70 (52.38)	58.9 (68.95)
DNMC‡	1.68 (3.85)	1.59 (4.92)	13.05 (10.48)	20.37 (21.44)
Total medical expenditure§	7.98 (12.83)	6.24 (11.88)	63.76 (54.26)	79.27 (74.38)

*Among 345 pneumonia cases who visited health facilities 18 had had a visit to a traditional healer with a mean (SD) cost of 1.72 (1.49). Among 341 diarrhoea cases who visited health facilities 16 had had a visit to a traditional healer with a mean (SD) cost of 2.74 (3.39).

†Other costs include expenses incurred for food, lodging, etc.

‡DMC includes registration fee, medicines, laboratory and diagnostics and bed charges while DNMC includes transport, lodging, traditional healer, etc.

§Total medical expenditure is the sum of DMC and DNMC.

DMC, direct medical costs; DNMC, direct non-medical costs.

The mean total medical expenditures for an episode of pneumonia, diarrhoea, severe pneumonia or severe diarrhoea were 2.3–3.8 times higher in private facilities than at government hospitals (table 4). Type of health facility visited was the main predictor of a difference in the mean total medical expenditure for each disease category. Child healthcare services were not entirely free of charge at public PHC facilities. At health posts, though consultation fees were not paid, parents were obliged to buy medication from private outlets because of a lack of drug stock at health posts. In most of the health centres, parents paid fees for consultation and medications.

**Table 4:** Average total medical expenditure per disease episode in US$ by type of health facility visited

Diagnosis	Type of health facility	No. of cases (%)	Mean cost (SD)
Pneumonia*	Health post (HP)	42 (14%)	1.61 (2.71)
	Health centre (HC)	181 (60%)	4.06 (5.91)
	Government hospital	57 (19%)	12.08 (12.05)
	Private clinic/hospital	23 (7%)	28.12 (8.85)
Diarrhoea*	Health post	47 (15%)	0.97 (1.97)
	Health centre	183 (59%)	3.89 (6.13)
	Government hospital	57 (19%)	5.66 (5.97)
	Private clinic/hospital	22 (7%)	21.41 (11.17)
Severe pneumonia with inpatient care	Health post	0	–
	Health centre	3 (7%)	12.13 (8.80)
	Government hospital	26 (62%)	47.89 (28.81)
	Private clinic/hospital	13 (31%)	139.66 (71.97)
Severe diarrhoea with inpatient care	Health post	0	–
	Health centre	1 (3%)	15.59
	Government hospital	20 (63%)	55.92 (58.96)
	Private clinic/hospital	11 (34%)	151.86 (84.33)

*For both conditions, medical costs per episode were five to seven times greater in private facilities compared with health centres. The differences by facility type were statistically significant for both conditions (p<0.001).

There were marked variations in total medical expenditures by wealth quintile, place of residence and region (tables 5 and 6). Table 5 shows the distribution of total medical expenses for diarrhoea and pneumonia by wealth quintile. The wealthiest households spent six times more on treatment as compared to the poorest households. The mean total medical expenditure was 1.7–2 times higher in urban than in rural households. Urban households and wealthier quintiles were more likely to visit private facilities or public hospitals than PHC facilities. 33% of urban households and 16% of rural households had outpatient visits in either private facilities or public hospitals. Similarly, 39% of the wealthiest two quintiles and 13% of the poorest two quintiles had outpatient visits in either private facilities or public hospitals. Number of severe pneumonia and severe diarrhoea cases was disproportionately high in Addis Ababa, accounting for 27% and 38% of all reported severe cases, respectively.

**Table 5:** Mean monthly consumption expenditure and total medical expenditure per disease episode in US$ by wealth quintile

	Pneumonia*		Diarrhoea*	
Wealth quintile	Mean monthly consumption expenditure (US$)	Mean total medical expenditure (US$)	Mean monthly consumption expenditure (US$)	Mean total medical expenditure (US$)
I	56	3.17 (6%)	48	3.18 (7%)
II	90	4.71 (5%)	89	4.58 (5%)
III	107	9.13 (9%)	115	4.84 (4%)
IV	125	8.20 (7%)	126	6.45 (5%)
V	209	15.11 (7%)	195	13.43 (7%)

*For both conditions, medical costs per episode were four to five times greater in the highest wealth quintile compared with the lowest. The difference by wealth quintile was statistically significant for both conditions (p<0.001). The numbers in parentheses denote the mean total medical expenditure divided by the mean monthly consumption expenditure.

**Table 6:** Total medical expenditure (mean and SD) per disease episode in US$ by place of residence and region

	Place of residence		Region				
Diagnosis	Urban	Rural	Amhara	SNNPR	Oromia	Tigray	Addis Ababa
No. of observations	411 (60%)	274 (40%)	125 (18%)	105 (15%)	196 (29%)	146 (21%)	114 (17%)
Pneumonia*	8.66 (12.58)	4.36 (6.35)	2.07 (1.60)	5.26 (4.08)	7.23 (10.58)	9.21 (10.53)	12.30 (18.74)
Diarrhoea*	6.51 (9.81)	2.99 (3.94)	2.36 (3.71)	3.73 (3.24)	4.55 (6.98)	6.65 (8.83)	7.97 (13.56)
Severe pneumonia	91.01 (75.52)	48.35 (39.06)	34.11 (12.85)	25.47 (9.02)	53.34 (35.83)	45.86 (15.66)	126.03 (77.34)
Severe diarrhoea	98.81 (83.40)	59.08 (78.75)	–	16.30 (3.37)	58.51 (54.60)	10.75	146.59 (82.53)

*For both conditions, medical costs per episode were three to six times greater in Addis Ababa compared with Amhara region. The regional differences were statistically significant for both conditions (p<0.002).

SNNPR, Southern Nations Nationalities and People Region.

### Catastrophic health expenditures and impoverishment

Household annual mean total expenditures and mean non-food expenditures were US$1320 and US$349, respectively. For outpatient care, 0.3–0.6% of households incurred CHE at 40% annual capacity to pay threshold level (table 7). The figure rises to 21–24% when we used the 40% monthly capacity to pay threshold level as the denominator. The incidence of CHE was higher for severe cases of pneumonia and diarrhoea. Disaggregation of CHE by place of residence and wealth quintile revealed that rural and poor households were less able to cope with any given level of health expenditure than urban and wealthier households (table 7). For outpatient pneumonia or diarrhoea episodes, 0.3% of households were pushed into extreme poverty due to OOP payments. The figures were much higher for inpatient care, where 7% and 6% of the households with severe pneumonia and severe diarrhoea cases, respectively, were pushed below the extreme PL.

**Table 7:** Incidence of Catastrophic Health Payments per disease episode, defined with respect to capacity to pay in Ethiopia, 2013

		Out-of-pocket health spending as share of CTP, at 40% threshold budget share							
		Annual CTP	Monthly CTP						
Diagnosis		Average	Average	Rural	Urban	The bottom half quintile	The upper half quintile	Private facilities	Public facilities
Both*	All categories	1.6% (631)	31% (631)	36% (286)	27% (345)†	35% (341)	26% (290)†	78% (64)	25% (567)†
Outpatient	Pneumonia	0.3% (277)	24% (277)	27% (103)	23% (174)	28% (159)	17% (118)†	83% (21)	19% (256)†
	Diarrhoea	0.6% (280)	21% (280)	31% (157)	15% (123)†	29% (150)	13% (130)†	53% (19)	19% (261)†
Inpatient*	Severe pneumonia or diarrhoea	11% (74)	96% (74)	100% (26)	94% (48)	100% (32)	93% (42)	96% (24)	96% (50)

*Both include outpatient and inpatient cases.

†The rate of catastrophic head count varied significantly by place of residence, type of health facility visited or wealth quintile. The numbers in parentheses are the number of observations.

CTP, capacity to pay.

## Discussion and conclusions

Our study documented OOP payments and time loss for the two most common causes of morbidity and mortality in children 0–59 months in Ethiopia. The findings demonstrate that OOP expenditures associated with diarrheal illness or pneumonia can be a substantial economic burden for households. Most of the total medical expenditures (ranging from 74% to 80%) consist of direct medical costs. Medications were the major contributor to direct medical costs for outpatient and inpatient visits, followed by bed charges for inpatient care. Several previous studies conducted elsewhere reported comparable estimates of total household medical expenditures, as well as identifying direct medical costs and medications as the major drivers of total medical expenditures.^9–11^ ^30–32^ Among the direct non-medical costs, transportation costs presented families with a significant financial hurdle even before accessing needed formal care.

OOP expenses varied depending on the facility visited, families spending significantly higher costs in private health facilities. The average OOP expenses for treating pneumonia and diarrhoea in private facilities were US$28 and US$21 per case, respectively. Households incurred the least costs at public PHC facilities, where the mean total medical expenditures at health centres for outpatient care of pneumonia or diarrhoea were US$4.1 and US$3.9, respectively.

Our study shows that wealthier households have greater demand and access to health services and a wider range of choices to select from. Urban and wealthier households were more likely to visit private facilities or public hospitals where the perceived quality of care is superior. At the same time, the poor have lower overall OOP expenses, especially for outpatient services, reflecting their more limited access to health services. Barnet and Tefera reported a preference among poor households in Ethiopia for higher-level health facilities because the quality and quantity of services available at PHC facilities were perceived as inferior.^33^ Despite such perceptions, poor households were less likely to visit facilities where they were more likely to incur higher expenditures, possibly a function of households' inability to absorb medical payments. User fees at public health facilities are associated with decreased service usage, even more so for marginalised segments of the population such as women, children and the poor.^7^ ^34^ ^35^ Evidence from similar settings in Africa also suggests that abolition of user fees results in increased service usage in all population groups.^36^ User fees could hamper the Ethiopian government's efforts to make essential priority services universally accessible.^37^ One of the fundamental impediments to UHC is over reliance on direct payments at the time people need care.^38^ ^39^

Our cost findings could be underestimation for two reasons: (1) we did not factor productivity loss into cost estimates, and (2) households may incur additional costs after 2 weeks of follow-up. Additionally, we failed to reach 16% of the households for follow-up interviews. Our study did not include cases of pneumonia or diarrhoea for which households did not seek care—and therefore did not incur any cost—or those that directly go to the pharmacy or visit traditional healers; these exclusions could lead to overestimation of the mean medical expenses and incidence of CHE at a population level. Facility exit polls only address health service users and are likely to be biased towards better-off individuals and urban residents. In our sample, only 5% of the households were below the extreme PL, well below the national figure. The high proportion of severe cases and the absence of health posts in Addis Ababa could inflate the mean cost of treatment for inpatient and outpatient pneumonia and diarrhoea cases in the region. Furthermore, we did not assess the source of funding for treatment episodes (eg, saving, borrowing, etc). Owing to the small number of inpatient cases of severe pneumonia and diarrhoea, the results of subgroup analyses should be interpreted with caution. Despite these limitations, the study has an important strength. Data on OOP expenses were collected immediately when incurred, thereby minimising recall bias.

Despite government efforts to increase access to preventive services, poor and rural households bear a considerable risk of CHE and impoverishment due to OOP medical expenses when seeking curative care for pneumonia and diarrhoea in Ethiopia. For these households, the increased risk of CHE could exacerbate the inequity and impoverishment that are already prevalent in Ethiopia.^40^ Ensuring financial risk protection is one of the health sector's objectives, as prescribed in the national health policy of Ethiopia.^41^ Achievement of this objective requires revisiting the existing health financing strategy for high priority services that place a substantial burden of payment on households at the point of service delivery.

## Footnotes

i Rural areas comprise all areas not classified as urban. Urban areas are defined as: (1) all administrative capitals (regional, zone and district capitals); (2) localities with urban dwellers association not included in (1); (3) all localities which are not included either in (1) or (2) above having a population of 1000 or more persons, and whose inhabitants are primarily engaged in non-agricultural activities.

ii AE is calculated using the formula: AE=(A+αK)^θ^, where A is the number of adults in the household, K is the number of children, α is the ‘cost of children’ and θ reflects the degree of economies of scale. On the basis of what was proposed by Deaton and Zaidi,^29^ we chose a value of 0.3 for α and 0.9 for θ, because food accounts for a large proportion of total consumption and economies of scale are relatively limited.
